# UV treatment of the digestive fluid of *Nepenthes hemsleyana* pitcher plants affects their digestive process, possibly via reducing microbial inquilines

**DOI:** 10.1007/s00442-025-05749-6

**Published:** 2025-06-24

**Authors:** Julien L. Bota, Christel Baum, Sofie Gawronski, T. Ulmar Grafe, Gerald Kerth, Michael G. Schöner, Caroline R. Schöner

**Affiliations:** 1 Applied Zoology and Nature Conservation, Zoological Institute and Museum, University of Greifswald, Loitzer Str. 26, 17489 Greifswald, Germany; 2 Soil Science, Faculty of Agricultural and Environmental Sciences, University of Rostock, Justus-Von-Liebig Weg 6, 18051 Rostock, Germany; 3 Faculty of Science, University Brunei Darussalam, Gadong, BE1410 Brunei Darussalam; 4 Present Address: WSL-Institute for Snow- and Avalanche Research SLF, Flüelastrasse 11, 7260 Davos Dorf, Switzerland

**Keywords:** Plant microbiota, Microbial ecology, Phytotelmata, Species interactions, Digestive mutualism

## Abstract

Interactions with microbes are ubiquitous, and many of them are essential for the survival and success of plants. In *Nepenthes* pitcher plants, they occur as part of a diverse community of organisms, so-called inquilines, that live inside the digestive fluid of the pitcher traps*.* However, evidence is ambiguous regarding the role of microbial inquilines: they may complement the plants’ prey digestion, fix atmospheric N, act as competitors that reduce plant-available nutrients or affect the plants in other ways unrelated to the breakdown of prey. In a field experiment on Borneo, we investigated the effect of UV disinfection of the digestive fluid on prey digestion of *N. hemsleyana* that captures and digests insects as well as bat faeces in its pitchers. We show that in the short term, the photosynthetic performance of plants with UV-treated digestive fluids decreases compared to untreated plants, likely due to lower abundances of microbial inquilines. However, at the end of 2 months, responses of pitcher plants with UV-treated and untreated digestive fluids tend to equalise. Nutrient source, whether from insects or bat faeces, does not influence prey digestion. We expect our findings to be a starting point for unveiling the ecological role of microbial inquilines in pitcher plants and how they interact with other inquiline groups of higher trophic levels. Ultimately, this will also help to improve understanding of the functioning and evolution of convergent interactions in other carnivorous plants.

**Supplementary Information:**

The online version supplementary material available at 10.1007/s00442-025-05749-6.

## Introduction

Interactions with microbes are ubiquitous and essential for the survival and success of plants: amongst other functions, they provide limiting nutrients (Van Der Heijden et al. 2008; Jacoby et al. 2017), mitigate biotic and abiotic stresses (Mendes et al. 2013) and promote plant growth (Vacheron et al. 2013). In pitcher plants, microbes occur as part of a highly diverse community of organisms, so-called inquilines, that live inside the pitcher traps of the plant genera *Sarracenia, Cephalotus* and *Nepenthes* (Adlassnig et al. 2011; Gray et al. 2012; Bittleston 2018; Bittleston et al. 2022). Whilst they appear to play an essential role in the digestion of captured prey in *Sarracenia* (Luciano and Newell 2017; Miller et al. 2018), their role in the digestive processes of *Cephalotus* and *Nepenthes* is less clear. So far, it has been suggested that they could complement the digestive processes of their host plants (Adlassnig et al. 2011; Bittleston et al. 2016, 2018; Chan et al. 2016; Bittleston 2018), affect them detrimentally by damaging the pitchers or reducing plant-available nutrients (Adlassnig et al. 2011; Bittleston 2018; Bittleston et al. 2022) or have no effect on prey digestion (Adlassnig et al. 2011).

**Fig. 1:**
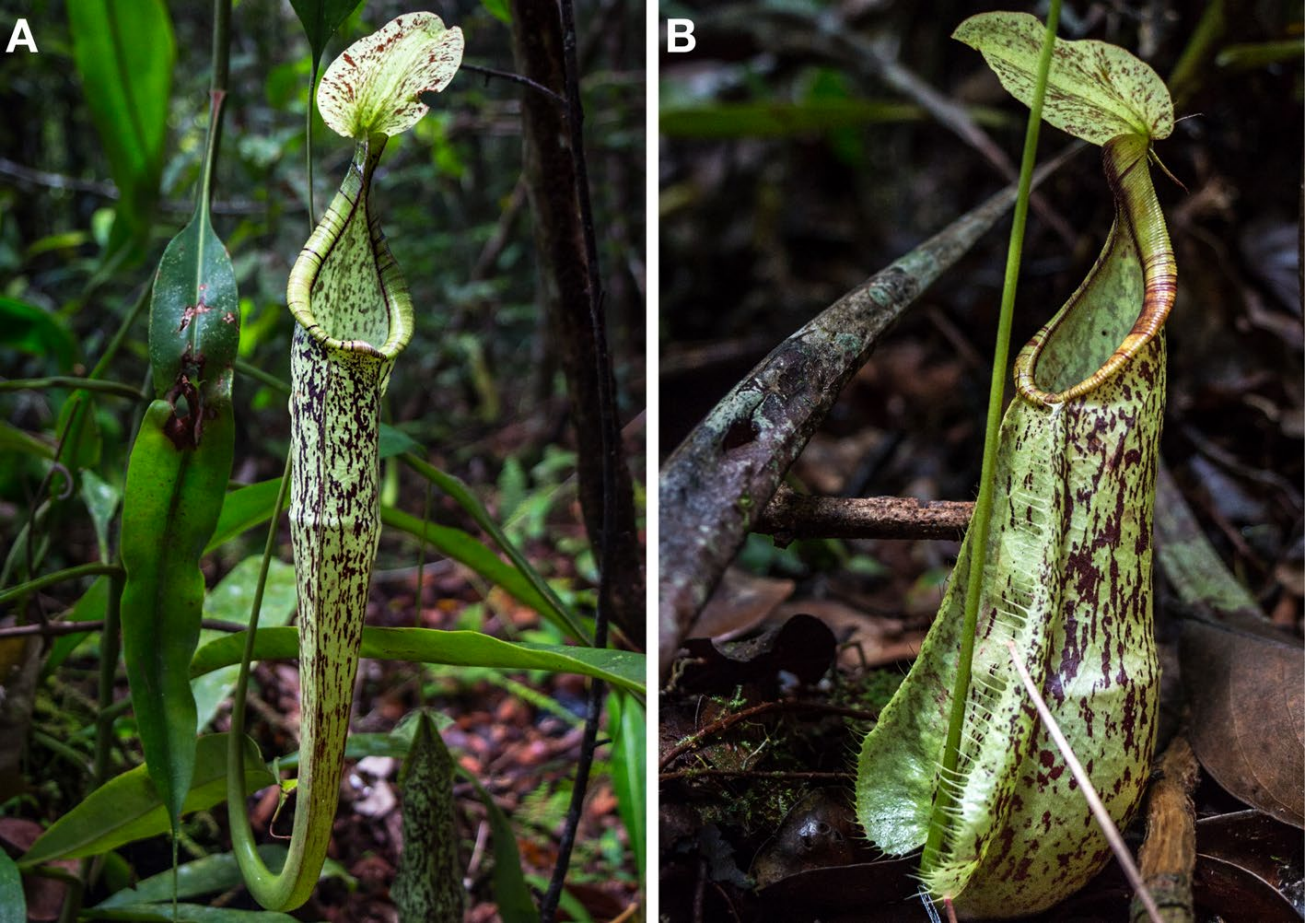
**A** Aerial and **B** lower pitcher of *N. hemsleyana* in its natural habitat on Borneo. Photos by Julien L. Bota

The pitcher plant family Nepenthaceae is distributed throughout the Paleotropics with centres of diversity in Borneo, Sumatra and the Philippines (Clarke and Moran 2016). Nutrient acquisition strategies of the different *Nepenthes* species are highly diverse: most species rely on insectivorous strategies (Moran 1996; Bauer et al. 2012; Clarke and Moran 2016) but some have diverged at least partially from purely carnivorous to detritivorous (Moran et al. 2003) and coprophagous lifestyles (Chin et al. 2010; Grafe et al. 2011). *Nepenthes hemsleyana* (Fig. 1) deploys a dual strategy whereby it captures arthropods (Moran 1996) but also faeces of the insectivorous bat species *Kerivoula hardwickii* that roosts inside its’ pitchers (Grafe et al. 2011). Capturing bat faeces allows *N. hemsleyana* to cover between 34 and 96% of its foliar nitrogen demand (Grafe et al. 2011; Schöner et al. 2016) and it benefits from increased growth and photosynthesis (Schöner et al. 2016). Whilst the upper part of *N. hemsleyana*’s pitcher body is involved in the attraction of mutualistic bats or insect prey (Schöner et al. 2015; Thorogood et al. 2018), the lower part is filled with a digestive fluid and contains several glands that secrete diverse enzymes for the digestion of arthropod prey or bat faeces (An et al. 2002; Rottloff et al. 2016; Matušíková et al. 2018; Kocáb et al. 2021).

Apart from its function for prey digestion, the digestive fluid also harbours a complex inquiline community of arthropods, vermiform organisms, rotifers and microbes that either colonise the pitcher opportunistically or are limited to them for at least stages of their life cycles (Bittleston 2018). The micro-inquiline community consists thereby of fungi, protists but also green algae that add organic carbon into the system which in turn is consumed by other inquilines. The most abundant and diverse organisms living in the pitcher fluid, however, are bacteria (Bittleston 2018). Amongst them are members of the Chitinophagaceae, which have been suggested to be gut inhabitants of *K. hardwickii*, as well as other taxa that are associated with the mammalian digestive tract, such as *Lactobacillus, Mobiluncus* and *Anaerococcus* (Sickel et al. 2016). Other gut symbionts of both vertebrates and insects occur in the fluids as well, though at low abundances (Sickel et al. 2016). The digestive fluid furthermore contains bacteria with nitrogen-fixing abilities such as *Bradyrhizobium, Methylosinus* and *Burkholderia* that belong to the Rhizobiales and Burkholderiales (Sickel et al. 2016). As common rhizosphere symbionts, they can promote plant growth through the fixation of atmospheric nitrogen and the production of phytohormones (Richardson et al. 2009). In fact, bat faeces may serve as substrate for those taxa and become further nitrogen enriched through microbial activities (Schöner et al. 2016). Conversely, also saprophytic taxa with urease and nitrate reduction capabilities are present such as *Mycobacterium, Corynebacterium* and *Sphingobacterium* (Shapleigh 2006, 2013; Sickel et al. 2016).

So far, it is unclear how the microbial inquiline community influences prey digestion of *Nepenthes* (Adlassnig et al. 2011; Bittleston 2018; Matušíková et al. 2018). On the one hand, microbes may act as decomposers that support the breakdown of prey through the excretion of extracellular digestive enzymes that complement the set of enzymes produced by the pitcher plant, which is for example assumed to be the case in the breakdown of chitin (Chan et al. 2016; Bittleston et al. 2018). Their capabilities further include breakdown of protein, starch, xylan, and cellulose as well as the enrichment of the digestive fluid with organic carbon and nitrogen (Chan et al. 2016; Bittleston 2018; Bittleston et al. 2023). On the other hand, microbial inquilines may compete with the plants for nutrients as also denitrifiers and saprophytes occur in the pitcher fluid (Sickel et al. 2016), though *Nepenthes* plants are not defenceless against detrimental microbes as they can modify the properties of the digestive fluid such as pH, viscosity and coloration (Gilbert et al. 2020). Furthermore, they express antimicrobial substances (Raj et al. 2011), enabling them, in combination with the modulation of fluid properties, to maintain species-specific microbial associations (Gilbert et al. 2020). It is also possible that the relationship with at least parts of the pitcher microbiome is of purely commensalistic or amensalistic nature (Adlassnig et al. 2011). However, the type of interaction with the pitcher’s microbiome during the digestive process remains elusive, and further research on this topic is required (Schöner et al. 2016; Sickel et al. 2016; Chan et al. 2016).

To explore the effect of the microbial inquiline community on prey digestion of *N. hemsleyana*, we investigated in the field whether plants with UV-treated digestive fluids perform inferiorly or superiorly to untreated ones. We predicted that unaffected microbial abundance in the digestive fluid would accelerate nutrient breakdown and thus support nutrient uptake by the plant. In addition, we also predicted that the nutrient source, i.e. insects versus bat faeces, would influence the effect of the UV treatment. Investigating the ecological role of microbial inquilines in *N. hemsleyana* will not only improve our understanding of interactions between microbes and other *Nepenthes* spp., but will also increase our general knowledge of the potential functions that microbes fulfil for plants.

## Materials and methods

### Study site and experimental setup

**Fig. 2:**
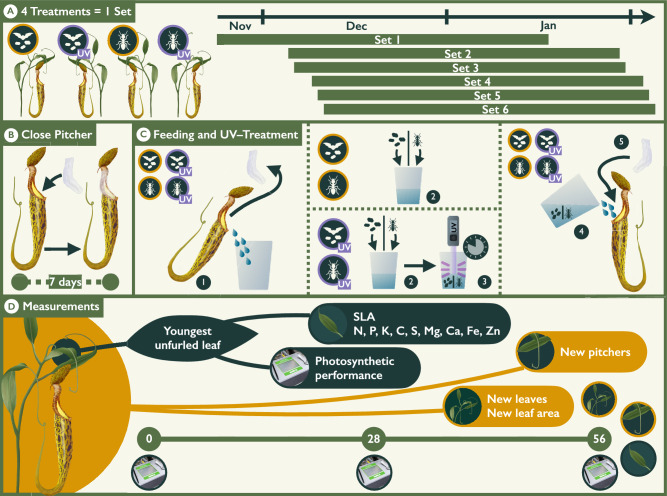
Experimental setup and measurements. **A** Selected plants were randomly assigned to one of four feeding treatments: bat faeces, arthropods, bat faeces + UV and arthropods + UV. A full set of treatments was always started simultaneously. **B** One week prior to the start of the treatments, pitcher orifices were sealed with plastic wrap to prevent the unintentional capture of prey. **C** To feed the pitchers, the plastic wrap was temporarily removed, and the pitcher fluid and contents were emptied into a receptacle (step 1). Based on the assigned feeding treatment, 6 mg of bat faeces or 5 mg of arthropods was added to the receptacle (2). Rainwater was added as needed to maintain the natural fluid level. For UV treatments, the pitcher fluid and the added prey items were irradiated using a portable UV disinfector for 48 s (3). The treated digestive fluid and prey items were then returned to the pitcher, and the orifice was resealed with plastic wrap (4). **D** Overview of measurements performed on the youngest unfurled leaf and the whole plant, along with their timing over the 56-day experimental period

Field experiments were performed in Mulu, Sarawak, Malaysian–Borneo during the rainy season between November 2017 and February 2018. Experimental plants grew at a single site with sandy soils located in a forested area in the lowland. Exact location data are withheld to protect the plant population at this site. We selected 24 *N. hemsleyana* plants that grew at least 2 m apart from each other and which possessed at least two young pitchers whose lids had opened within the last 24 h. Due to a lack of suitable aerial pitchers, we also had to include lower pitchers (Fig. 1B) that do not accommodate roosting bats. Selected pitchers were randomly assigned to two treatment groups where pitchers were either provided with arthropods or bat faeces as prey items. In a second step, half of the plants of each feeding treatment were randomly selected for an UV treatment to reduce the abundance of microbes in the pitchers’ digestive fluid (Supplement S 1.1). As available plants were limited, it was not possible to start all replicates at once. Therefore, the plants were assigned to six sets containing replicates of all four treatments (faeces, arthropods, faeces + UV, arthropods + UV) that were started simultaneously (Fig. 2A). To prevent pitchers from capturing arthropods or bat faeces, we closed all pitcher orifices of the selected plants with plastic wrap 1 week prior to the beginning of the experiment (Fig. 2B, Supplement S1.3 for methodological discussion of pitcher sealing). We then fed the two youngest pitchers of each plant on a daily basis over a period of 8 weeks, with the natural capture rates of 6 mg bat faeces or 5 mg arthropod prey per day as determined by Schöner et al. (2016). In two cases where one of the two fed pitchers was damaged (faeces + UV and arthropods + UV), we transferred the contents and fluid from the damaged pitcher to the undamaged one and continued the experiment with only one pitcher. Due to limited availability of faeces from *Nepenthes hemsleyana*’s mutualistic bat partner *Kerivoula hardwickii*, fresh faeces from other local insectivorous bat species were used instead. We collected guano once from a single location without further homogenisation beneath a large roosting colony in Dear Cave, Mulu. Whilst it was not possible to determine the exact species composition of the colony at the collection site, *Chaerephon plicata* is known to be the most abundant species at the cave (Lundberg et al. 2022). Fed arthropods were collected in the surroundings of the experimental site and consisted mostly of ants and termites which are also captured naturally by *N. hemsleyana* (Moran 1996). To facilitate the feeding procedure, we emptied the digestive fluids and contents of the two selected pitchers into a plastic receptacle daily, where the respective prey items were added (Fig. 2C). If required, UV-treated rainwater was added to maintain the natural level of the digestive fluid. Before the digestive fluid was reintroduced into the pitchers, pitcher fluids of plants in the UV treatments were treated with a portable UV disinfector for 48 s (SteriPEN Ultra, Katadyn Produkte AG, Kemptthal, Switzerland; Supplement S1.1 and S1.2 for efficiency of the treatment and possible side effects on enzyme activity). After the digestive fluid and its prey contents were reintroduced into the pitcher, we closed the pitcher orifice again with plastic wrap to avoid any macro-inquilines or unintended prey capture (Fig. 2C).

### Measurements of photosynthetic performance

To acquire information about the photosynthetic performance of the plants, we measured chlorophyll a fluorescence of the youngest fully developed leaf at days 0, 28 and 56 with a portable photosynthesis yield analyser (MINI-PAM II, Heinz Walz GmbH, Effeltrich, Germany; Fig. 2D). Fluorescence measurements give information about the extent to which energy absorbed by chlorophyll is used in photosystem II (PSII) or excess energy damages the photosynthetic apparatus. Although photosynthesis is not measured directly, inference about the plants’ photosynthetic performance is possible by measuring relative electron transport rates (ETR) that approximate the rate of electrons flowing through the photosynthetic chain and thus correlate with carbon fixation (Maxwell and Johnson 2000; Ralph and Gademann 2005). Before the measurement, leaves were darkened for 20 min using a leaf clip (Leaf Clip DLC-8, Heinz Walz GmbH) to close all reaction centres during dark adaptation. The PAM was set to an average measuring light intensity of ≈ 0.06 μmol m^−2^ s^−1^, an actinic light intensity of 190 μmol m^−2^ s^−1^ and saturation pulse intensity of 2,000 μmol m^−2^ s^−1^ whereby each pulse lasted 0.6 s. At first, we measured minimum fluorescence (F_0_) and after applying a saturation pulse also maximum fluorescence (F_m_) was measured. After 40 s, actinic illumination was turned on and an induction curve with 12 saturation pulses every 60 s was obtained allowing the measurement of minimum fluorescence levels of the illuminated sample (F_0_’), maximum fluorescence levels of the illuminated sample (F_M_’) and momentary fluorescence levels of the illuminated sample (F) shortly before or after the saturation pulses. Actinic light was switched off for 30 s at the end of the induction curve and a rapid light curve (RLC) was initiated, increasing actinic light (PAR) from an initial actinic illumination intensity of 65 μmol m^−2^ s^−1^ in eight steps lasting 10 s each to 1,150 μmol m^−2^ s^−1^. At the end of each irradiation interval, a saturation pulse was given and F_0_’, F_M_’ and F were measured. RLCs measure ETR in response to increasing PAR, allowing assessment of photosynthetic performance over a wide range of ambient light intensities (Ralph and Gademann 2005). As photosynthetic performance changes throughout the day, measurements were always conducted around mid-day to ensure comparability. Using the WinControl-3 Software (v. 3.29, Heinz Walz GmbH, Effeltrich, Germany), we calculated maximum quantum yield (F_v_/F_m_ as defined by Kitajima and Butler 1975), quantum yield of photosystem II (Φ_PSII_; Genty et al. 1989) and relative electron transfer rates (ETR) with a leaf absorptance constant of 0.84 and PSI/PSII allocation factor of 0.5. To quantitatively compare RLCs, we fitted the data to the equation derived by Platt et al. (1980):

1 $$ETR={ETR}_{mPot}*\left(1-{e}^{\frac{\alpha *PAR}{{ETR}_{mPot}}}\right)*{e}^{\frac{\beta *PAR}{{ETR}_{mPot}}}$$

where α represents the initial slope of the RLC in the light-limited region, β characterises the downturn of the curve after reaching ETR_max_, and ETR_mPot_ is the maximum photosynthetic output the sample could sustain in the absence of photoinhibition. We further estimated the maximum electron transport rate (ETR_max_), photoinhibition index (I_b_), and the minimum irradiance where electron transport is saturated (I_k_) using the following equations:

2 $${ETR}_{max }= {ETR}_{mPot}*\left(\frac{\alpha }{\alpha + \beta }\right)*{\left(\frac{\beta }{\alpha + \beta }\right)}^{\frac{\beta }{\alpha }}$$

3 $${I}_{b}={ETR}_{mPot}/\beta$$

4 $${I}_{k}={ETR}_{max}/\alpha$$

In addition, by integrating the curve across all measured PAR intensities, we attempted to capture all these parameters in one that represents the overall performance of the plants (ETR_total_).

As heavy rain disturbed PAM measurements of Set 4 on day 28 of the field experiment, we had to exclude measurements conducted on this day from the analysis.

### Elemental analyses

We marked the youngest fully unfurled leaf of each pitcher plant when treatments were started and harvested it at the end of the experimental period to determine total concentrations of carbon, nitrogen and sulphur with a CNS-Analyzer (Vario EL, Fa. Foss Heraeus, Hanau, Germany; Fig. 2D) using air-dried and milled plant material (DIN ISO 10694: 1996–08). The elemental concentration of further nutrients in plant material was determined after digesting 0.1 g biomass with 5 mL HNO_3_ and 3 mL H_2_O_2_ in a microwave (Mars Xpress, CEM, Kamp-Lintfort, Germany) followed by diluting with H_2_O dest. to a volume of 25 mL (DIN 38406-E22). Concentrations of P, K, Mg, Ca, Fe and Zn were measured with inductively coupled plasma optical emission spectrometry (ICP-OES) at wavelengths of 214.914 nm, 766.490 nm, 285.213 nm, 317.933 nm, 238.204 nm, and 206.200 nm, respectively.

### Measurement of plant growth

We counted the number of newly developed pitchers (> 0.5 cm in length) and the number of newly developed leaves at the end of the experiment (Fig. 2D). To identify possible changes in the photosynthetic area of the plants, we determined the cumulative leaf surface area of all fully developed new leaves at the end of the experiment. Subsequently, we harvested the youngest fully developed leaf, dried it on silica gel, determined its dry weight and calculated the specific leaf area (Hunt 2017).

### Data analysis

We performed all analyses in R v. 4.3.2 and v. 4.4.2 (R Core Team 2024) and RStudio v. 2024.12.0.467 (Posit team 2024). To prepare our raw data for statistical analysis, we used the R-package “dplyr” v. 1.1.4 (Wickham et al. 2023). Rapid light curves were fitted using non-linear least squares regression with the Levenberg–Marquardt algorithm using the R-package “minpack.lm”, v. 1.2–1 (Elzhov et al. 2023). To test for significant treatment effects influencing photosynthesis, foliar nutrient contents and plant growth, we used linear mixed-effects models. These analyses were conducted using the R-package “lme4” v. 1.1–35.5 (Bates et al. 2015) and the package “lmerTest” v. 3.1–3 (Kuznetsova et al. 2017) for analysis of variance (ANOVA Type III). We assessed normality, homogeneity of variance and outliers for each model by examining residual vs. fitted plots and normal q–q plots using the R-package “performance” v. 0.12.4 (Lüdecke et al. 2021). To improve the model characteristics, log- and inverse transformation was applied where required (Supplement S1.4). We incorporated prey type, UV treatment, and, in the case of PAM measurements, sampling day as interacting fixed effects. Non-significant interaction terms were dropped for model simplification following the approach described by Zuur et al. (2009) using lmerTest’s “step”-function. To account for the blocking of simultaneously started treatments in sets over the course of the experiment, set identity was added as a random factor. As PAM measurements were conducted repeatedly over different sampling days, we further included plant identity as a random effect in these models. Marginal and conditional R^2^ (R^2^_m_, R^2^_c_) were calculated to estimate the explained variance by fixed effects alone and in combination with random effects using the R-package “MuMIn”, v. 1.48.4 (Bartoń 2024). We obtained fitted values and standard errors from our models using the R-package “effects” v. 4.2–2 (Fox and Weisberg 2018a, b). To analyse counted pitchers and leaves in the field experiment, we used a Poisson regression model in combination with analysis of deviance using the R-package “car” v. 3.1–3 (Fox and Weisberg 2018a). We checked model specifications by simulating scaled residuals and assessed residual distribution in a q–q plot as well as homogeneity of residuals in a residual vs. fitted plot. In addition, we tested models for over- and under-dispersion using the R-package “DHARMa”, v. 0.4.7 (Hartig 2024). Alpha was set to p < 0.05, and significant effects were further analysed post-hoc by contrasting estimated marginal means using the R-package “emmeans”, v.1.10.5 (Lenth 2024). False discovery rate was controlled using the Benjamini–Hochberg method. We created all plots using the R-packages ggplot2 v. 3.5.1 (Wickham 2016) and cowplot v. 1.1.3 (Wilke 2024).

## Results

### Photosynthetic performance

**Fig. 3:**
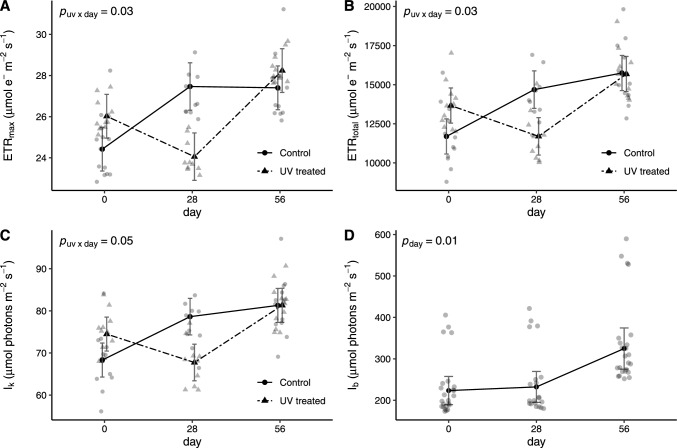
Temporal effects of digestive fluid sterilisation on photosynthetic performance of the youngest fully unfurled leaf in *N. hemsleyana,* measured using rapid light curves with a portable photosynthesis yield analyser. **A** Maximum electron transport rate (ETR_max_), **B** cumulative electron transport rates across all measured PAR levels (ETR_total_), (C) minimum irradiance (I_k_) at which electron transport is saturated, and (D) photoinhibition index (I_b_), reflecting the PAR intensity required to inhibit potential ETR. Displayed are model predictions ± standard errors, with fitted values shown in the background

Over the course of the experiment, maximum electron transport rates (ETR_max_) of *N. hemsleyana* were significantly influenced by the UV treatment of the digestive fluid, regardless of the type of prey fed (*p*_*uv ⨯ day*_ = 0.03, Tab. 1, Fig. 3A). In plants with untreated digestive fluids, ETR_max_ showed a non-significant tendency to increase from 24.42 ± 1.06 (mean ± SE) to 27.47 ± 1.15 µmol e^−^ m^−2^ s^−1^ within the first 28 days of the experiment (Fig. 3A; day 0 vs. 28: *t*(41.9) = -2.24, *p* = 0.08). In contrast, responses were delayed in plants where digestive fluids were treated with UV, with ETR_max_ showing a significant upward trend only after the first 28 days of the experiment from 24.06 ± 1.15 to 28.24 ± 1.06 µmol e^−^ m^−2^ s^−1^ (Fig. 3A; day 28 vs. 56: *t*(41.9) = -3.1, *p* = 0.03). At the end of the experimental period, however, final ETR_max_ of plants with UV-treated and untreated digestive fluids reached comparable levels (Fig. 3A).

**Table 1:** ANOVA results for linear mixed-effects models assessing photosynthetic performance of *N. hemsleyana*. Displayed are the single effects of fed prey type (“Prey”), fluid sterilisation (“UV”), sampling day (“Day”) as well as their interactions on maximum electron transport rate (ETR_max_), cumulative electron transport rates across all measured PAR levels (ETR_total_), minimum irradiance (I_k_) at which electron transport is saturated, and photoinhibition index (I_b_). Marginal R^2^ (R^2^_m_) gives the explained variance of fixed effects, and conditional R^2^ (R^2^_c_) the explained variance of fixed and random effects of the respective model

	ETR_max_	ETR_total_	I_k_	I_b_
	*F *^*p*^	*F *^*p*^	*F *^*p*^	*F *^*p*^
Prey	0.1 ^0.78^	0.2 ^0.64^	0.7 ^0.43^	- 0.3 ^0.59^
UV	0.1 ^0.75^	0.1 ^0.72^	0.2 ^0.7^	0 ^0.89^
Day	**4.5 **^**0.02**^	**7.6 **^**0.002**^	**5.5 **^**0.01**^	**5.1 **^**0.01**^
Prey ⨯ UV	–	–	–	–
Prey ⨯ Day	–	–	–	–
UV ⨯ Day	**3.9 **^**0.03**^	**4.0 **^**0.03**^	**3.3 **^**0.05**^	*–*
Prey ⨯ UV ⨯ Day	–	–	–	–
R^2^_m_	0.16	0.18	0.15	0.10
R^2^_c_	0.37	0.49	0.45	0.39

This response was further reflected by total ETR (ETR_total_) that quantifies cumulative ETR measured over all PAR intensities of the RLC (*p*_*uv ⨯ day*_ = 0.03, Tab. 1, Fig. 3B). Within the first 28 days of the experiment, ETR_total_ of plants in which the digestive fluid was not UV treated showed a non-significant increasing trend from 11,691.65 ± 1,122.56 to 14,684.24 ± 1,197.98 µmol e^−^ m^−2^ s^−1^ (day 0 vs. 28: *t*(41.4) = -2.4, *p* = 0.06) with final values on day 56 of 15,746.55 ± 1,122.56 µmol e^−^ m^−2^ s^−1^ that were significantly higher compared to the beginning of the experiment on day 0 (*t*(40.1) = -3.47, *p* = 0.01). In contrast, ETR_total_ of plants with UV-treated digestive fluids only increased significantly from 11,697.24 ± 1,197.98 on day 28 to 15,681.69 ± 1,122.56 µmol e^−^ m^−2^ s^−1^ on day 56 (Fig. 3B; *t*(41.4) = -3.2, *p* = 0.01). The minimum saturating irradiance of electron transport (I_k_) showed similar tendencies in its responses to the UV treatment of the digestive fluid (Fig. 3C; Tab. 1). The photoinhibition index I_b_ which gives the PAR intensity required to inhibit potential ETR increased significantly from 232.25 ± 37.24 on day 28 to 324.88 ± 49.63 µmol photons m^−2^ s^−1^ on day 56 (*t*(43.0) = -2.47, *p* = 0.03) regardless of the treatment of the digestive fluid (Fig. 3D).

### Nutrient content of leaves

**Fig. 4:**
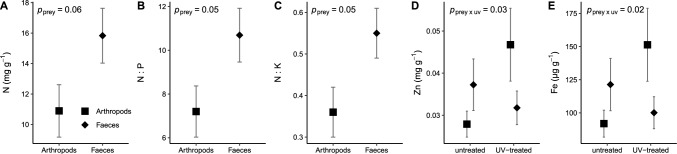
**A**–**C** Effects of feeding treatments on foliar N content, and interactive effects of fluid sterilisation and feeding treatment on **D** Zn and **E** Fe content of the youngest fully unfurled leaf, measured at the end of the 56-day experimental period. Displayed are mean values ± standard errors

Foliar N content of *N. hemsleyana* showed a non-significant trend to be 45% higher in plants that were fed with faeces compared to arthropod-fed ones (Fig. 4A, Table 2: main effect “prey”: *F*(1) = 3.9, *p* = 0.06). This was further reflected by a 48% higher N:P ratio (Fig. 4B, Table 2: main effect “prey”: *F*(1) = 4.2, *p* = 0.05) and a 53% higher N:K ratio in faeces-fed plants compared to those fed with arthropods (Fig. 4C, Table 2: main effect “prey”: *F*(1) = 4.2, *p* = 0.05).

**Table 2:** ANOVA results from linear mixed-effects models assessing foliar nutrient content in the youngest fully unfurled leaf of *N. hemsleyana*, measured at the end of the 56-day experimental period

	N	N:P	N:K	Zn	Fe
	F ^*p*^	F ^*p*^	F ^*p*^	F ^*p*^	F ^*p*^
Prey	*3.9 *^*0.06*^	**4.2 **^**0.05**^	**4.2 **^**0.05**^	0.0 ^0.89^	0.1 ^0.77^
UV	2.1 ^0.17^	*3.2 *^*0.09*^	2.2 ^0.16^	1.5 ^0.24^	1.1 ^0.32^
Prey ⨯ UV	–	*–*	–	**5.5 **^**0.03**^	**6.0 **^**0.02**^
R^2^_m_	0.22	0.26	0.23	0.25	0.25
R^2^_c_	0.22	0.26	0.23	0.25	0.25

Shown are the single effects of fed prey type (“Prey”), fluid sterilisation (“UV”), and their interaction on N, N:P, N:K, Zn and Fe. Marginal R^2^ (R^2^_m_) gives the explained variance of fixed effects, and conditional R^2^ (R^2^_c_) the explained variance of fixed and random effects of the respective model

Zn was significantly influenced by the interaction of UV treatment and fed prey type (Tab. 2: “prey ⨯ uv”: *F*(1) = 5.5, *p* = 0.03), whereby it showed a non-significant trend to increase by 68% in arthropod-fed plants when the digestive fluid was UV treated (Fig. 4D; *t*(14) = 2.6, *p* = 0.09). Similarly, Fe was significantly influenced by the interaction of UV treatment and fed prey type (Tab. 2: “prey ⨯ uv”: *F*(1) = 6, *p* = 0.02), whereby Fe content of arthropod-fed plants showed a non-significant tendency to increase by 65% when the fluid was UV treated (Fig. 4E; *t*(14) = 2.5, *p* = 0.1).

### Plant growth

The number of newly developed leaves, cumulative leaf area, specific leaf area (SLA) and the formation of new pitchers were not significantly influenced by the feeding and UV treatments.

## Discussion

In this study, we investigated how UV treatment of the digestive fluid of *N. hemsleyana* affects the digestion of captured prey. In addition, we examined whether these processes are influenced by the type of prey. Though the responses exhibited considerable variability, we found evidence of detrimental effects of the UV treatment in the short term, but not in the long term. Observed dynamics of the UV treatment were not influenced by the type of captured prey, except for Fe and Zn uptake in plants that were fed with arthropods.

Foliar nutrient contents measured at the end of the feeding period showed that *N. hemsleyana* plants were limited by N availability (critical values given in Osunkoya et al. 2007: N < 20 mg/g, N:P < 14, N:K < 2.1). This holds true for most *Nepenthes* species and has been found for *N. hemsleyana* before (Osunkoya et al. 2007). Even though the plants were not released from N-deficiency throughout our experiment, photosynthetic performance improved when we fed them with arthropods or faeces as observed in previous studies (Pavlovič et al. 2009; Schöner et al. 2016). Moreover, plants where the digestive fluid was not treated with UV showed an improved photosynthetic performance in response to feeding compared to plants treated with UV (indicated by increased ETR_max_, ETR_total_, I_k_ and I_b_ within the first 28 days of the experiment). In plants with UV-treated digestive fluid, improvements in photosynthetic performance were delayed and equal levels were only reached towards the end of the feeding period after 56 days, which suggests that nutrient uptake during the first 28 days was not as efficient as when digestive fluids were untreated.

UV-C light has a high capacity to inactivate or destroy microbes by damaging their DNA or RNA and is, therefore, widely applied for water disinfection (Gray 2014). The UV-Steripen that we used for our UV treatment has been shown to reduce the abundance of protozoa, bacteria and viruses between 94.98% and 99.99% in 1 l of water after 90 s of irradiation (Timmermann et al. 2015). Although microbes exhibit varying sensitivities to UV radiation (Gray 2014; Timmermann et al. 2015) and we only irradiated the digestive fluid and its prey contents, it is likely that our daily UV treatment reduced microbial abundance in the digestive fluid. Subsequent experiments with similarly treated digestive fluids of *Nepenthes* plants from the greenhouse support this assumption (Supplement S1.1). However, we have no validation of the effectivity of the UV treatment from the field and lack information about the abundance, composition and possible changes to the microbial community over time. Considering the hypotheses of Chan et al. (2016) and Bittleston (2018), that microbial inquilines act as decomposers and support prey digestion of *Nepenthes*, it seems likely, though, that the improved photosynthetic performance of untreated plants was caused by different abundances of microbes in the digestive fluid. Previous studies about the microbiome in *Nepenthes* pitchers have identified several species that may complement the digestive capabilities of the plants through the breakdown of protein, starch, xylan, chitin and cellulose (Chan et al. 2016; Bittleston et al. 2018) but also possibly through N-fixation (Schöner et al. 2016; Sickel et al. 2016; Bittleston et al. 2023) or their role as primary producers (Bittleston 2018). Microbial inquiline communities of the Nepenthaceae also show similarities in their composition and richness to those of the convergently evolved pitcher plant families Cephalotaceae and Sarraceniaceae (Bittleston et al. 2018, 2022). At least for *Sarracenia purpurea* it is known that microbial inquilines play a crucial role in the digestion of captured prey by producing enzymes for prey degradation (Gray et al. 2012; Luciano and Newell 2017; Miller et al. 2018). We can only speculate about the reasons for why the photosynthetic performance of UV-treated and untreated plants aligned towards the end of the experiment: though *Nepenthes* plants are usually able to influence the composition of microbial communities by regulating crucial abiotic factors in their pitchers (Gilbert et al. 2020), the microbial community composition in untreated pitchers may have changed over time from taxa that support the breakdown of prey in favour of other ones that reduce nitrate or urease, e.g. Mycobacterium, Corynebacterium or Sphingobacterium. Such taxa have been identified in the digestive fluids of *N. hemsleyana* (Sickel et al. 2016). In addition, saprophytes may incorporate the nutrients, making them temporarily unavailable (Sickel et al. 2016), or other biotic or abiotic factors may become limiting. Alternatively, it would also be possible that UV-resistant pathogens benefitted from the UV treatment and inhibited photosynthesis. Subsequent pathogen-related reactions of the plants then could have induced enhancement of photosynthesis in the long term (Selvaraj and Fof 2012).

Over the observation period, however, the effects on photosynthesis were not reflected in plant growth or the nutrient content of the leaves. This could indicate that (a) the observed effects on photosynthesis were not crucial for the plant, (b) effects may only become visible in the long term or (c) were overshadowed by other factors as the experimental setup considered only microbial inquilines. Under natural conditions, pitchers are colonised by a whole multitude of different macro-inquilines, which represent many different taxa and include all trophic levels (Bittleston 2018) but were excluded in our experimental setup. Their presence may change the here observed outcome fundamentally: prey breakdown may be supported by higher trophic levels, e.g. through shredding of carcasses (Miller et al. 2018), but nutrients may also get ultimately lost when species that only spend their larval stages inside the pitcher finally emerge (Bittleston 2018). Under such conditions of strong nutrient competition, a delayed uptake, as observed in plants with UV-treated digestive fluids and presumably reduced microbial inquiline abundance, would be detrimental, whilst accelerated exploitation of captured prey, promoted by microbial inquilines, could be of great benefit.

N content of bat guano from Deer Cave used for feeding is 7.6% (Lundberg et al. 2022). In contrast, arthropods have a higher N content that ranges between 8.1% for herbivores and 12.6% for predators (Fagan et al. 2002). Despite this, foliar N content was higher in faeces-fed plants, regardless of UV treatment. This is consistent with experiments of Schöner et al. (2016) who found that bat faeces constitute a superior nutrient source for *N. hemsleyana*. Unlike arthropod prey, where nitrogen is mainly bound in protein, chitin and nucleic acids, bat faeces and especially urine contain a high proportion of urea that can be taken up comparably fast by the plants, but also uric acid, ammonia, creatinine and chitin (Schöner et al. 2016; Kocáb et al. 2021).

It is important to note that our conclusions about a possible microbial contribution to prey digestion in this study are speculative. For a full assessment of the interactions between the pitcher plant microbiome and its host plant, information on direct effects of the UV treatment on diversity, abundance, dynamics and functional composition of the microbial communities is required. Future studies should include the diverse macro-inquiline community as it can profoundly influence the net outcome of the interactions (Kneitel and Miller 2002; Miller et al. 2018). In addition, more profound effects of microbial inquilines on plant growth and health may only become visible over longer experimental periods, and the here considered experimental period of 8 weeks might have been too short. Although we did not account for environmental conditions within individual pitchers, future studies may benefit from including variables such as digestive fluid biochemistry, pH, viscosity, and pitcher volume as covariates, as these factors can influence microbial community composition and dynamics (Bittleston et al. 2020, 2023; Gilbert et al. 2020). Despite the limitations of our study, we believe that it may be a starting point to investigate the interactions between the microbial inquiline communities and their *Nepenthes* host plants.

## Conclusion

We conclude that the observed differences in the photosynthetic performance of *N. hemsleyana* in response to a UV treatment following feeding of the pitchers with either arthropods or bat faeces were most likely caused by differences in the abundance of microbial inquilines in the digestive fluid. These possibly support the breakdown of captured prey and complement the plant’s own enzymes. Plant responses to the UV treatment were not affected by the type of captured prey, except for arthropod-fed plants with UV-treated digestive fluids which showed an elevated Zn and Fe uptake.

Pitcher plants are promising model systems to address digestive mutualisms with microbes as different nutrient sequestration strategies that involve multispecies interactions evolved several times also in distantly related *Nepenthes* taxa and are further mirrored in the three unrelated plant families of pitcher plants *Nepenthaceae*, *Sarraceniaceae* and *Cephalotaceae* (Bittleston et al. 2016). Comparative and experimental studies of such interactions that emerged independently and share similar physiological or ecological functions—so-called convergent interactions (Bittleston et al. 2016)—will help to investigate the ecological role of microbes in the digestive process of pitcher plants as well as how these interactions evolved (Ellison and Gotelli 2001; Bittleston et al. 2016, 2018).

## Supplementary Information

Below is the link to the electronic supplementary material.

Supplementary material 1 (PDF 35525 KB)

## Data Availability

Data publicly available on dryad: DOI: 10.5061/dryad.00000009p

## Code availability

Publicly available on dryad: DOI: 10.5061/dryad.00000009p

## Declarations

### Research permits

Permit Nos. WL91/2017 and NPW.907.4.4(Jld.14)-190 issued by the Sarawak Forestry Department.

## References

[CR1] Adlassnig W, Peroutka M, Lendl T (2011) Traps of carnivorous pitcher plants as a habitat: composition of the fluid, biodiversity and mutualistic activities. Ann Bot 107:181–194. 10.1093/aob/mcq238 21159782 10.1093/aob/mcq238 PMC3025736

[CR2] An C-I, Fukusaki E-I, Kobayashi A (2002) Aspartic proteinases are expressed in pitchers of the carnivorous plant Nepenthes alata Blanco. Planta 214:661–667. 10.1007/s004250100665 11882933 10.1007/s004250100665

[CR3] Bartoń K (2024) MuMIn: Multi-Model Inference. R package version 1.48.4. https://CRAN.R-project.org/package=MuMIn

[CR4] Bates D, Mächler M, Bolker B, Walker S (2015) Fitting linear mixed-effects models using lme4. J Stat Softw 67:1–48. 10.18637/jss.v067.i01

[CR5] Bauer U, Di Giusto B, Skepper J, Grafe TU, Federle W (2012) With a flick of the lid: a novel trapping mechanism in nepenthes gracilis pitcher plants. PLoS ONE 7:e38951. 10.1371/journal.pone.0038951 22719998 10.1371/journal.pone.0038951 PMC3374759

[CR6] Bittleston LS, Pierce NE, Ellison AM, Pringle A (2016) Convergence in multispecies interactions. Trends Ecol Evol 31:269–280. 10.1016/j.tree.2016.01.006 26858111 10.1016/j.tree.2016.01.006

[CR7] Bittleston LS, Wolock CJ, Yahya BE, Chan XY, Chan KG, Pierce NE, Pringle A (2018) Convergence between the microcosms of Southeast Asian and North American pitcher plants. Elife 7:e36741. 10.7554/eLife.36741 30152327 10.7554/eLife.36741 PMC6130972

[CR8] Bittleston LS, Gralka M, Leventhal GE, Mizrahi I, Cordero OX (2020) Context-dependent dynamics lead to the assembly of functionally distinct microbial communities. Nat Commun 11:1440. 10.1038/s41467-020-15169-0 32188849 10.1038/s41467-020-15169-0 PMC7080782

[CR9] Bittleston LS, Benson EL, Bernardin JR, Pierce NE (2022) Characterization and comparison of convergence among Cephalotus follicularis pitcher plant-associated communities with those of Nepenthes and Sarracenia found worldwide. Front Plant Sci 13:887635. 10.3389/fpls.2022.887635 35734258 10.3389/fpls.2022.887635 PMC9207445

[CR10] Bittleston LS, Wolock CJ, Maeda J, Infante V, Ané J-M, Pierce NE, Pringle A (2023) Carnivorous Nepenthes pitchers with less acidic fluid house nitrogen-fixing bacteria. Appl Environ Microbiol 89:e0081223. 10.1128/aem.00812-23 37338413 10.1128/aem.00812-23 PMC10370301

[CR11] Bittleston LS (2018) Commensals of Nepenthes pitchers. In: Ellison A, Adamec L (eds) Carnivorous Plants: Physiology, ecology, and evolution. Oxford University Press, pp 314–332. 10.1093/oso/9780198779841.003.0023

[CR12] Chan X-Y, Hong K-W, Yin W-F, Chan K-G (2016) Microbiome and Biocatalytic Bacteria in Monkey Cup (Nepenthes Pitcher) Digestive Fluid. Sci Rep 6:20016. 10.1038/srep20016 26817720 10.1038/srep20016 PMC4730220

[CR13] Chin L, Moran JA, Clarke C (2010) Trap geometry in three giant montane pitcher plant species from Borneo is a function of tree shrew body size. New Phytol 186:461–470. 10.1111/j.1469-8137.2009.03166.x 20100203 10.1111/j.1469-8137.2009.03166.x

[CR14] Clarke C, Moran JA (2016) Climate, soils and vicariance - their roles in shaping the diversity and distribution of Nepenthes in Southeast Asia. Plant Soil 403:37–51. 10.1007/s11104-015-2696-x

[CR15] Ellison AM, Gotelli NJ (2001) Evolutionary ecology of carnivorous plants. Trends Ecol Evol 16:623–629. 10.1016/S0169-5347(01)02269-8

[CR16] Elzhov TV, Mullen KM, Spiess A-N, Bolker B (2023) minpack.lm: R Interface to the Levenberg-Marquardt Nonlinear Least-Squares Algorithm Found in MINPACK, Plus Support for Bounds. R package version 1.2–4. https://CRAN.R-project.org/package=minpack.lm

[CR17] Fagan WF, Siemann E, Mitter C et al (2002) Nitrogen in insects: implications for trophic complexity and species diversification. Am Nat 160:784–802. 10.1086/343879 18707465 10.1086/343879

[CR18] Fox J, Weisberg S (2018a) An R companion to applied regression, 3rd edn. SAGE Publications, Thousand Oaks, CA

[CR19] Fox J, Weisberg S (2018) Visualizing fit and lack of fit in complex regression models with predictor effect plots and partial residuals. J Stat Softw 87:1–27. 10.18637/jss.v087.i09

[CR20] Genty B, Briantais JM, Baker NR (1989) The relationship between the quantum yield of photosynthetic electron transport and quenching of chlorophyll fluorescence. BBA-Gen Subjects 990:87–92. 10.1016/S0304-4165(89)80016-9

[CR21] Gilbert KJ, Bittleston LS, Tong W, Pierce NE (2020) Tropical pitcher plants (Nepenthes) act as ecological filters by altering properties of their fluid microenvironments. Sci Rep 10:4431. 10.1038/s41598-020-61193-x 32157122 10.1038/s41598-020-61193-x PMC7064508

[CR22] Grafe TU, Schöner CR, Kerth G et al (2011) A novel resource-service mutualism between bats and pitcher plants. Biol Lett 7:436–439. 10.1098/rsbl.2010.1141 21270023 10.1098/rsbl.2010.1141 PMC3097880

[CR23] Gray SM, Akob DM, Green SJ, Kostka JE (2012) The bacterial composition within the Sarracenia purpurea model system: local scale differences and the relationship with the other members of the food web. PLoS ONE 7:e50969. 10.1371/journal.pone.0050969 23227224 10.1371/journal.pone.0050969 PMC3515446

[CR24] Gray NF (2014) Ultraviolet Disinfection. In: Microbiology of Waterborne Diseases. Elsevier, pp 617–630. 10.1016/B978-0-12-415846-7.00034-2

[CR25] Hartig F (2024) DHARMa: Residual Diagnostics for Hierarchical (Multi-Level / Mixed) Regression Models. R package version 0.4.7. https://CRAN.R-project.org/package=DHARMa

[CR26] Hunt R (2017) Growth Analysis, Individual Plants. In: Thomas B, Murray BG, Murphy DJ (eds) Encyclopedia of Applied Plant Sciences, 2nd edn. Academic Press, Oxford, pp 421–429 10.1016/B978-0-12-394807-6.00226-4

[CR27] Jacoby R, Peukert M, Succurro A, Koprivova A, Kopriva S (2017) The Role of Soil Microorganisms in Plant Mineral Nutrition-Current Knowledge and Future Directions. Front Plant Sci 8:1617. 10.3389/fpls.2017.01617 28974956 10.3389/fpls.2017.01617 PMC5610682

[CR28] Kitajima M, Butler WL (1975) Quenching of chlorophyll fluorescence and primary photochemistry in chloroplasts by dibromothymoquinone. BBA - Bioenergetics 376:105–115. 10.1016/0005-2728(75)90209-1 1125215 10.1016/0005-2728(75)90209-1

[CR29] Kneitel JM, Miller TE (2002) Resource and top-predator regulation in the pitcher plant (Sarracenia purpurea) inquiline community. Ecology 83:680. 10.2307/3071873

[CR30] Kocáb O, Bačovčinová M, Bokor B et al (2021) Enzyme activities in two sister-species of carnivorous pitcher plants (Nepenthes) with contrasting nutrient sequestration strategies. Plant Physiol Biochem 161:113–121. 10.1016/j.plaphy.2021.01.049 33581619 10.1016/j.plaphy.2021.01.049

[CR31] Kuznetsova A, Brockhoff PB, Christensen RHB (2017) LmerTest package: Tests in linear mixed effects models. J Stat Softw 82:1–26. 10.18637/jss.v082.i13

[CR32] Lenth RV (2024) emmeans: Estimated Marginal Means, aka Least-Squares Means. R package version 1.10.5. https://CRAN.R-project.org/package=emmeans

[CR33] Luciano CS, Newell SJ (2017) Effects of prey, pitcher age, and microbes on acid phosphatase activity in fluid from pitchers of Sarracenia purpurea (Sarraceniaceae). PLoS ONE 12:e0181252. 10.1371/journal.pone.0181252 28719666 10.1371/journal.pone.0181252 PMC5515422

[CR34] Lüdecke D, Ben-Shachar M, Patil I et al (2021) Performance: An R package for assessment, comparison and testing of statistical models. J Open Source Softw 6:3139. 10.21105/joss.03139

[CR35] Lundberg J, McFarlane D, Van Rentergem G (2022) The nitrogen dynamics of Deer Cave, Sarawak, and the role of bat caves as biogeochemical sinks in Tropical Moist Forests. Int J Speleol 51:205–221. 10.5038/1827-806x.51.3.2438

[CR36] Matušíková I, Pavlovič A, Renner T (2018) Biochemistry of prey digestion and nutrient absorption. In: Ellison A, Adamec L (eds) Carnivorous Plants: Physiology, ecology, and evolution. Oxford University Press, pp 207–220 10.1093/oso/9780198779841.003.0016

[CR37] Maxwell K, Johnson GN (2000) Chlorophyll fluorescence – a practical guide. J Exp Bot 51:659–668. 10.1093/jexbot/51.345.659 10938857 10.1093/jxb/51.345.659

[CR38] Mendes R, Garbeva P, Raaijmakers JM (2013) The rhizosphere microbiome: significance of plant beneficial, plant pathogenic, and human pathogenic microorganisms. FEMS Microbiol Rev 37:634–663. 10.1111/1574-6976.12028 23790204 10.1111/1574-6976.12028

[CR39] Miller TE, Bradshaw WE, Holzapfel CM (2018) Pitcher-plant communities as model systems for addressing fundamental questions in ecology and evolution. In: Ellison A, Adamec L (eds) Carnivorous Plants: Physiology, ecology, and evolution. Oxford University Press, pp 333–348. 10.1093/oso/9780198779841.003.0024

[CR40] Moran JA (1996) Pitcher dimorphism, prey composition and the mechanisms of prey attraction in the Pitcher Plant Nepenthes rafflesiana in Borneo. J Ecol 84:515–525. 10.2307/2261474

[CR41] Moran JA, Clarke CM, Hawkins BJ (2003) From carnivore to detritivore? Isotopic evidence for leaf litter utilization by the tropical pitcher plant Nepenthes ampullaria. Int J Plant Sci 164:635–639. 10.1086/375422

[CR42] Osunkoya OO, Daud SD, Di-Giusto B et al (2007) Construction costs and physico-chemical properties of the assimilatory organs of Nepenthes species in Northern Borneo. Ann Bot 99:895–906. 10.1093/aob/mcm023 17452380 10.1093/aob/mcm023 PMC2802909

[CR43] Pavlovič A, Singerová L, Demko V, Hudák J (2009) Feeding enhances photosynthetic efficiency in the carnivorous pitcher plant Nepenthes talangensis. Ann Bot 104:307–314. 10.1093/aob/mcp121 19454591 10.1093/aob/mcp121 PMC2710902

[CR44] Platt T, Gallegos C, Harrison WG (1980) Photoinhibition of photosynthesis in natural assemblages of marine phytoplankton. J Mar Res 38:687–701

[CR45] Posit team (2024) RStudio: Integrated Development Environment for R. Version 2024.12.0.467. Posit Software, PBC, Boston, MA. http://www.posit.co/

[CR46] R Core Team (2024) R: A Language and Environment for Statistical Computing. R Foundation for Statistical Computing, Vienna, Austria. https://www.r-project.org

[CR47] Raj G, Kurup R, Hussain AA, Baby S (2011) Distribution of naphthoquinones, plumbagin, droserone, and 5-O-methyl droserone in chitin-induced and uninduced Nepenthes khasiana: Molecular events in prey capture. J Exp Bot 62:5429–5436. 10.1093/jxb/err219 21862483 10.1093/jxb/err219

[CR48] Ralph PJ, Gademann R (2005) Rapid light curves: A powerful tool to assess photosynthetic activity. Aquat Bot 82:222–237. 10.1016/j.aquabot.2005.02.006

[CR49] Richardson AE, Barea JM, McNeill AM, Prigent-Combaret C (2009) Acquisition of phosphorus and nitrogen in the rhizosphere and plant growth promotion by microorganisms. Plant Soil 321:305–339. 10.1007/s11104-009-9895-2

[CR50] Rottloff S, Miguel S, Biteau F, Nisse E, Hammann P, Kuhn L, Chicher J, Bazile V, Gaume L, Mignard B, Hehn A, Bourgaud F (2016) Proteome analysis of digestive fluids in Nepenthes pitchers. Ann Bot 117:479–495. 10.1093/aob/mcw001 26912512 10.1093/aob/mcw001 PMC4765550

[CR51] Schöner MG, Schöner CR, Simon R, Grafe TU, Puechmaille SJ, Ji LL, Kerth G (2015) Bats Are Acoustically Attracted to Mutualistic Carnivorous Plants. Curr Biol 25:1911–1916. 10.1016/j.cub.2015.05.054 26166777 10.1016/j.cub.2015.05.054

[CR52] Schöner CR, Schöner MG, Grafe TU, Clarke CM, Dombrowski L, Tan MC, Kerth G, Bonser S (2016) Ecological outsourcing: a pitcher plant benefits from transferring pre-digestion of prey to a bat mutualist. J Ecol 105:400–411. 10.1111/1365-2745.12653

[CR53] Selvaraj K, Fof B (2012) An overview of plant photosynthesis modulation by pathogen attacks. In: Advances in Photosynthesis - Fundamental Aspects. InTech. 10.5772/27124

[CR54] Shapleigh JP (2006) The Denitrifying Prokaryotes. In: Falkow S, Rosenberg E, Schleifer K-H, Stackebrandt E (eds) The Prokaryotes, 3rd edn. Springer, New York, New York, NY, pp 769–792

[CR55] Shapleigh JP (2013) Denitrifying Prokaryotes. In: Rosenberg E, DeLong EF, Lory S, et al. (eds) The Prokaryotes. Springer Berlin Heidelberg, Berlin, Heidelberg, pp 405–425. 10.1007/978-3-642-30194-0

[CR56] Sickel W, Grafe TU, Meuche I, Steffan-Dewenter I, Keller A (2016) Bacterial Diversity and Community Structure in Two Bornean Nepenthes Species with Differences in Nitrogen Acquisition Strategies. Microb Ecol 71:938–953. 10.1007/s00248-015-0723-3 26790863 10.1007/s00248-015-0723-3

[CR57] Thorogood CJ, Bauer U, Hiscock SJ (2018) Convergent and divergent evolution in carnivorous pitcher plant traps. New Phytol 217:1035–1041. 10.1111/nph.14879 29131340 10.1111/nph.14879

[CR58] Timmermann LF, Ritter K, Hillebrandt D, Küpper T (2015) Drinking water treatment with ultraviolet light for travelers – Evaluation of a mobile lightweight system. Travel Med Infect Dis 13:466–474. 10.1016/j.tmaid.2015.10.005 26616307 10.1016/j.tmaid.2015.10.005

[CR59] Vacheron J, Desbrosses G, Bouffaud M-L, Touraine B, Moënne-Loccoz Y, Muller D, Legendre L, Wisniewski-Dyé F, Prigent-Combaret C (2013) Plant growth-promoting rhizobacteria and root system functioning. Front Plant Sci 4:356. 10.3389/fpls.2013.00356 24062756 10.3389/fpls.2013.00356 PMC3775148

[CR60] Van Der Heijden MGA, Bardgett RD, Van Straalen NM (2008) The unseen majority: Soil microbes as drivers of plant diversity and productivity in terrestrial ecosystems. Ecol Lett 11:296–310. 10.1111/j.1461-0248.2007.01139.x 18047587 10.1111/j.1461-0248.2007.01139.x

[CR61] Wickham H, François R, Henry L, et al (2023) dplyr: A Grammar of Data Manipulation. R package version 1.1.4. https://CRAN.R-project.org/package=dplyr

[CR62] Wickham H (2016) Ggplot2: Elegant graphics for data analysis, 2nd edn. Springer International Publishing, Basel, Switzerland 10.1007/978-0-387-98141-3

[CR63] Wilke CO (2024) cowplot: Streamlined Plot Theme and Plot Annotations for “ggplot2.” R package version 1.1.3. https://CRAN.R-project.org/package=cowplot

[CR64] Zuur AF, Ieno EN, Walker N, et al (2009) Mixed effects models and extensions in ecology with R. Springer New York, New York, NY. 10.1007/978-0-387-87458-6

