# Supplementary material for: UV treatment of the digestive fluid of Nepenthes hemsleyana pitcher plants affects their digestive process, possibly via reducing microbial inquilines
Source: Oecologia. 2025 Jun 24;207(7):108. doi: 10.1007/s00442-025-05749-6 (PMC12187847; doi:10.1007/s00442-025-05749-6)
Supplement: Supplementary file 1 — Supplementary material 1 (PDF 35525 KB) [file 442_2025_5749_MOESM1_ESM.pdf]

UV-treatment of the digestive fluid of *Nepenthes hemsleyana* pitcher plants affects their digestive process, possibly via reducing microbial inquilines

– Supplement –

S1.1 Testing the efficiency of the UV-sterilisation treatment

According to the manufacturer of the UV-Steripen we used in our experiment to reduce microbial abundance (SteriPEN Ultra, Katadyn Produkte AG, Kemptthal, Switzerland), it kills about 99.9% of all microbes present in a liquid[1]. To get an idea of the performance of the UV-Steripen in our experiment, we fed four pitchers of *N. hemsleyana* and closely related *N. rafflesiana* in a greenhouse of the Greifswald Botanical Garden (average temperature: 25.42 °C, relative humidity: 71.23 %) with arthropods or bat faeces. Twenty-four hours later, we emptied the pitcher fluid into a receptacle and treated it with a portable UV disinfector for 48 seconds (SteriPEN Ultra) and took a 100 µl sample of the digestive fluid. Samples were then plated on LB agar plates and incubated at 30°C for 24 h.

We then visually compared colony numbers of the sterilised samples with the unsterilised samples. This showed us that the culturable bacteria were greatly reduced by the UV-treatment (Fig. S1). Since complete eradication of the microbial communities would have been neither possible nor necessary (rapid recolonisation, pitchers can't be sterilised completely, sensitive field sites), this method appeared to be suitable for our purpose.

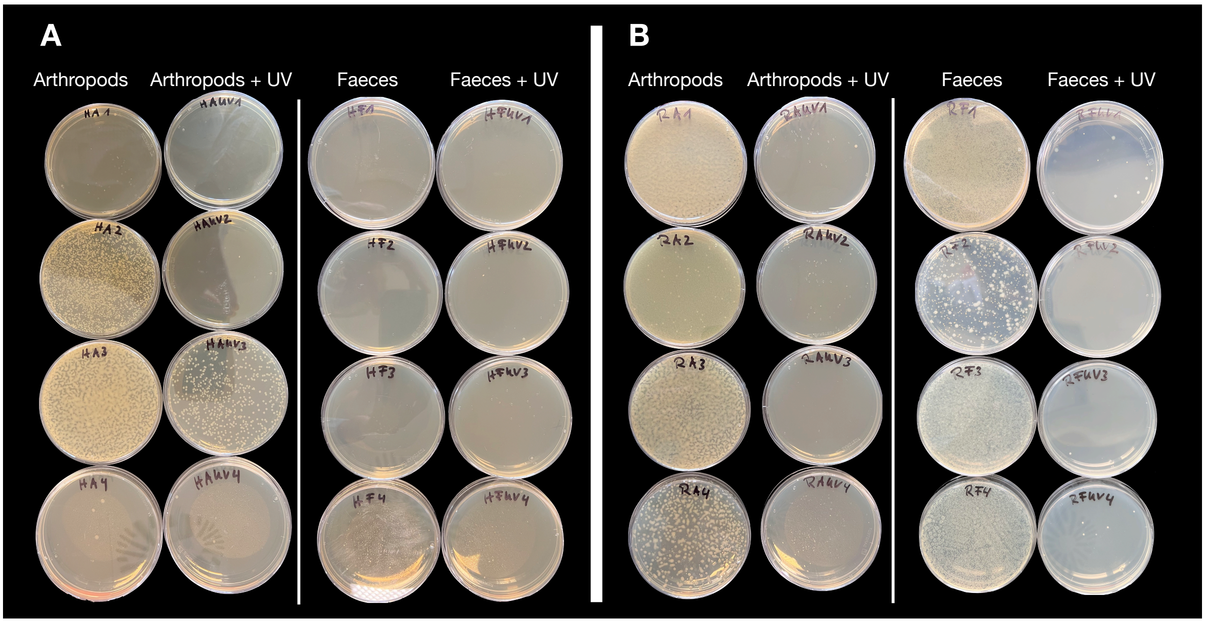

Fig. S1: LB Plates with fluid samples of (A) *N. hemsleyana* and (B) *N. rafflesiana* after incubation. Treatments are indicated above each column.

[1] Steripen Ultra UV Wasserentkeimer Wasseraufbereitung Outdoor. In: Katadyn Shop Deutschland. https://eu.katadyngroup.com/search?sSearch=+Steripen+Ultra+UV+Wasserentke. Accessed 24 Oct 2022

S1.2 Influence of UV-sterilisation on enzyme activity

To test for potential negative effects of the UV-sterilisation of the digestive fluid on enzyme activity, we compared protease activity of sterilised and non-sterilised digestive fluids of *N. hemsleyana*. We selected twelve pitchers from the greenhouse (average temperature: 25.42 °C, relative humidity: 71.23 %) and fed half of them with *Drosophila hydeii* and the other half with bat faeces. Additionally, we selected 3 pitchers for an unfed control. After 24 h we emptied the pitcher content into a plastic cup and took a sample of the non-sterilised digestive fluid. Subsequently, we sterilised the remaining digestive fluid with an UV-steriliser for 48 seconds (SteriPEN Ultra, Katadyn Produkte AG, Kemptthal, Switzerland) and took a second fluid sample.

We transferred samples immediately to the lab where we mixed 150 µl of each fluid sample with 250 µl Azocsassein (1 % w/v, solved in 50 mM Tris-HCl, pH 7.5) which releases an azo dye when degraded by proteases. Samples were then incubated for 6 h at 37°C until we terminated the reaction by adding 1.2 ml 10 (w/v) TCA. After 25 min incubation time, samples were centrifuged for 15 min with 12.300 rpm. Subsequently, we transferred 600 µl of the supernatant into a cuvette where we mixed it with 750 µl 1 M NaOH. After measuring the blank value, we measured absorption at 440 nm spectrophotometrically. We considered thereby the intensity of sample coloration proportional to the activity of proteases as they dissociate the azo dye from the cassein.

We analysed the absorbance data using a Kruskal-Wallis Rank Sum Test in R. Absorbance did not differ significantly between sterilised and non-sterilised digestive fluids of pitchers (p = 0.84; Fig. S2) which suggests that UV-sterilisation of the digestive fluid does not influence enzyme activity negatively.

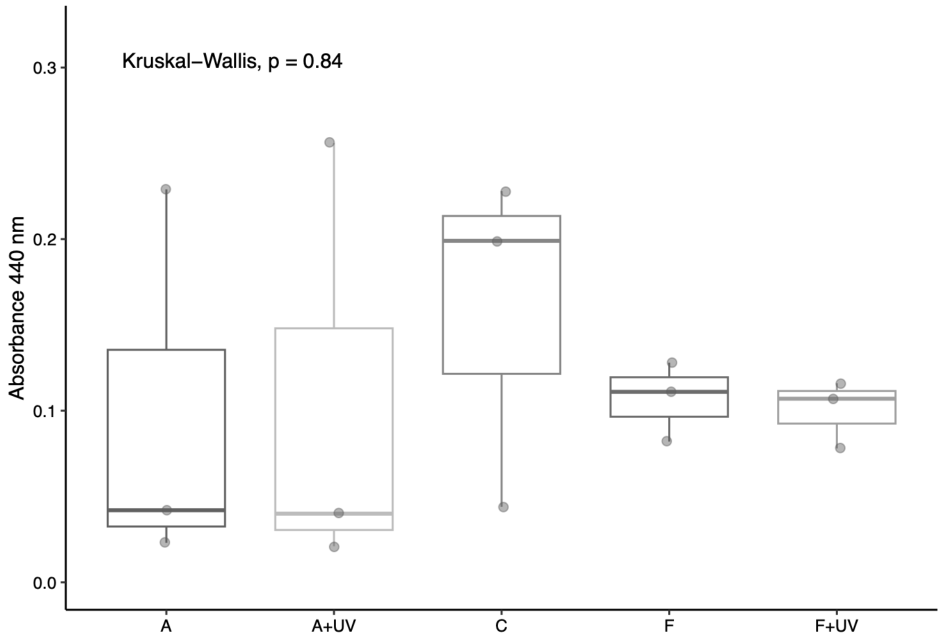

Fig. S2: Absorbance of the azo dye solved in the fluid samples of *N. hemsleyana* pitchers fed with Arthropods (A) or Faeces (F) and UV-sterilisation treatments (+ UV) as well as unfed control (C) at the end of the azocassein assay. Absorbance is proportional to activity of proteases, thus higher values indicate higher enzyme activity.

S1.3 Methodological considerations for prey exclusion

To prevent pitchers from capturing arthropods or bat faces, we closed all pitcher orifices of the selected plants with plastic wrap one week prior to the beginning of the experiment (Fig. S3 A). This approach was previously used by Schöner et al. in a field and greenhouse study[2] and proved effective in excluding external nutrient input into *N. hemsleyana* pitchers, such as small insects. However, this method also restricts airflow into the pitcher, which could influence the composition or dynamics of the initial microbial community. In later experiments, we opted for transparent organza bags (Fig. S3 B) to prevent prey capture while allowing airflow – an alternative that may have been more appropriate for this study as well. Despite this methodological limitation, we still observed clear responses to the UV-treatment.

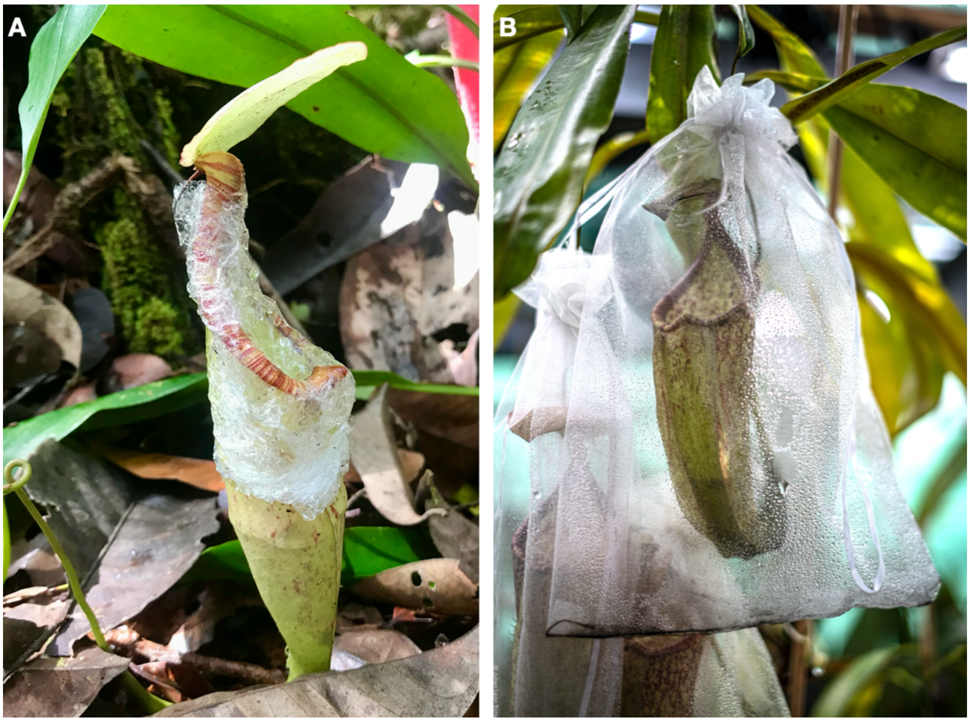

Fig. S3: (A) Plastic wrap sealing used in the field experiment with *N. hemsleyana*, (B) transparent organza bag used in later experiments to prevent prey capture of *N. rafflesiana*. Photos by Julien L. Bota

S1.4 Transformations of response variables

Tab. S1: Transformations of response variables used to improve model diagnostics.

Transformation	Transformed variables
Log-transformation	Ib, ETR_{tot}
Inverse	Zn, Fe

[2] Schöner CR, Schöner MG, Grafe TU, et al (2016) Ecological outsourcing: a pitcher plant benefits from transferring pre-digestion of prey to a bat mutualist. J Ecol 105:400–411
